# Nutrition as a Cultural-Biological Regulator of Ageing: The Concept of Ethnonutrigerontology

**DOI:** 10.7759/cureus.103502

**Published:** 2026-02-12

**Authors:** Aleksandr Martynenko

**Affiliations:** 1 Department of Internal Medicine, LLC “Multifunctional Medical Center” M-Clinic, Tashkent, UZB

**Keywords:** cultural diets, esthetic longevity, ethno-medicinal wisdom, food and nutrition, health aging, longevity medicine, traditional foods

## Abstract

Nutrition is one of the most modifiable determinants of ageing and longevity. However, most contemporary studies in nutrigerontology focus on universal biological mechanisms and often overlook cultural variations in dietary behavior. The aim of this conceptual review is to propose and theoretically substantiate Ethnonutrigerontology (ENuG), a newly coined interdisciplinary framework that conceptualizes nutrition as a cultural-biological regulator of ageing by linking culturally patterned dietary models to biological ageing mechanisms and later-life functional resilience. Unlike broader biocultural ageing frameworks, ENuG explicitly positions nutrition as the primary mediating mechanism connecting cultural systems to ageing biology. A conceptual synthesis of 60 peer-reviewed publications (1999-2025) was conducted using literature retrieved from PubMed, Scopus, and Web of Science. This study was designed as a theory-building conceptual synthesis rather than a systematic review or meta-analysis. Sources addressing the interrelation between nutrition, culture, and ageing were thematically analysed to identify structural gaps between biomedical and anthropological approaches. ENuG integrates three analytical levels - cultural-anthropological, biomedical, and gerontological - and formulates the causal sequence “Culture → Nutrition → Biology → Ageing → Functional resilience” as a heuristic model. Traditional diets, such as those of Okinawa, the Caucasus, and the Andean regions, have been associated in the literature with characteristics consistent with geronto-adaptive patterns, including metabolic balance, lower inflammatory burden, and favorable longevity indicators within their ecological and cultural contexts. As a proposal framework, ENuG is intended to generate testable hypotheses and guide future mixed-method empirical research rather than to provide causal proof.

## Introduction and background

Nutrition is one of the key modifiable factors associated with the rate of biological ageing processes, the development of chronic diseases, and the maintenance of functional reserves in later life [1]. Several systematic reviews emphasise that diets rich in fruits, vegetables, whole grains, fish, nuts, and unsaturated fats are associated with more favorable markers of ageing - including reduced inflammation, lower oxidative stress, and an improved cardiometabolic profile [2]. Bojang et al. (2023) and Kassis et al. (2023) highlight that nutrition in adulthood remains an important determinant of health, being associated with reduced risk of sarcopenia, metabolic disorders, and cognitive decline [3,4]. At the same time, an emerging discipline - nutrigerontology - investigates how nutrients, dietary patterns, and food-related behaviors influence the mechanisms of ageing and the risk of age-related diseases. The classical paper by Verburgh et al. (2014) contributed to establishing the foundations of this field [5]. Nevertheless, an analytical gap can be observed in the existing literature: most studies either focus on generalised “healthy” diets or on mechanistic pathways of ageing, yet rarely consider culturally specific dietary models - how ethnicity, tradition, climate, and ecology shape dietary features, and how these in turn may be associated with variation in ageing trajectories across populations [6-8].

In the context of globalisation, urbanisation, and the accelerated nutrition transition, many indigenous and traditional diets are undergoing significant transformation, which may be associated with alterations in dietary composition and the modification of previously established nutritional patterns, potentially affecting age-related resilience [9,10]. In this regard, it becomes particularly important to develop a conceptual framework capable of integrating biomedical approaches with the cultural and ecological dimensions of nutrition in ethnically diverse populations. The purpose of this article is to propose Ethnonutrigerontology (ENuG) as a newly coined term and conceptual framework. ENuG does not aim to replace biocultural gerontology; rather, it specifies nutrition as a distinct bridging mechanism that is often treated only as a background exposure in biocultural accounts. By making the causal pathway explicit (“Culture → Nutrition → Biology → Ageing → Functional resilience”), ENuG delineates a focused analytical space at the intersection of nutrigerontology, ethnonutrition, and ethnogerontology.

The central research question is: How do ethnocultural dietary models influence the biological mechanisms of ageing?

The proposed conceptual framework of ENuG seeks to bridge the gap between biomedical and cultural gerontology, providing a foundation for a truly biocultural analysis of ageing.

## Review

Materials and methods

This paper represents a conceptual review aimed at formulating and theoretically substantiating Ethnonutrigerontology (ENuG) as a distinct analytical framework. The approach synthesizes interdisciplinary sources encompassing gerontology, nutrition science, cultural anthropology, the sociology of ageing, and the ethnoecology of food.

This manuscript does not constitute a systematic or scoping review conducted under formal reporting guidelines (e.g., Preferred Reporting Items for Systematic Reviews and Meta-Analyses (PRISMA)). Rather, it represents a structured conceptual synthesis designed for theory development and integrative model construction.

Source Selection

The objective of the selection process was to identify publications containing both empirical data on nutrition and ageing, and conceptual approaches to their interpretation. Literature searches were conducted in PubMed, Scopus, and Web of Science for the period 1999-2025, with a focus on the most recent decade (2015-2025). The final search was conducted in August 2025, and searches were limited to publications in English. The following English keywords and their combinations were used: “nutrition and ageing”, “nutrigerontology”, “ethnonutrition”, “cultural food practices”, “ethnogerontology”, “traditional diets”, “longevity”, “biocultural ageing”.

Inclusion criteria comprised explicit linkage between nutrition and ageing; identification of cultural or ethnic characteristics; and publication in peer-reviewed journals. Explicit linkage was defined as the presence of biological ageing markers, age-related health outcomes, or mechanistic ageing pathways discussed in relation to dietary patterns. Cultural or ethnic characteristics were defined as reference to identifiable population groups, traditional food systems, or culturally patterned dietary behaviors. Studies lacking a description of cultural context were excluded. Additional consideration was given to conceptual and methodological papers addressing biocultural adaptation, sustainable nutrition, and the global nutrition transition. The initial search returned a large number of records (n = 59,603). Following relevance screening and conceptual eligibility assessment, 60 publications were selected to inform the theoretical synthesis. Selection was based on conceptual relevance to the analytical objectives rather than journal ranking or citation metrics. Documents from various international organizations were also used.

To enhance transparency, the synthesis followed a staged workflow: database search using predefined keyword combinations; title/abstract screening for explicit links between nutrition and ageing plus cultural/ethnic contextualization; full-text eligibility assessment; and inductive thematic coding to extract recurring constructs (e.g., cultural dietary rules, dietary rhythm/commensality, nutrition transition, and ageing-related biological pathways). Because the goal was conceptual integration rather than effect-size estimation, no meta-analysis or formal reporting checklist was applied.

Conceptual-Analytical Method

Unlike traditional systematic reviews that focus on quantitative synthesis of evidence, this study applied a conceptual synthesis approach, commonly used in the development of emerging scientific frameworks [11]. The primary objective was not statistical aggregation but the identification of structural gaps between existing disciplines - nutrigerontology, ethnonutrition, and ethnogerontology - and the construction of an integrative framework capable of linking their principles. The analysis employed theoretical coding and categorisation techniques, which included: Identifying key concepts (e.g., “nutrition as a mediator of ageing”, “cultural adaptation”, “traditional diets”); Comparing these concepts across disciplinary contexts; Developing a conceptual network scheme reflecting the integration of cultural, ecological, and biomedical factors in ageing processes. Themes were derived inductively through iterative reading and comparison across disciplines, and conceptual consistency was assessed through repeated cross-referencing between sources and emerging analytical categories.

The outcome was a synthetic conceptual framework of ENuG, encompassing three analytical levels: Cultural-anthropological: traditions, norms, and food practices; Biomedical: molecular and physiological mechanisms of ageing; Gerontological: functional outcomes and quality of life in later years.

Methodological Principles

The study is grounded on the following principles: interdisciplinarity: integration of theories and data from gerontology, nutrition science, ethnography, and sociology; contextuality: analysis of how local cultural environments and food ecologies influence ageing mechanisms; cultural sensitivity: recognition that dietary and healthy-ageing recommendations must respect cultural codes, symbolism, and identity [12]; systemic approach: consideration of ageing as an interaction among biological, social, and ecological systems, in line with the biocultural ageing model [13].

Limitations

The present study is conceptual and theory-building in nature; therefore, several limitations should be explicitly acknowledged.

First, ENuG is derived exclusively from secondary literature and does not incorporate primary empirical data. The proposed framework should thus be regarded as hypothesis-generating rather than confirmatory. As with any conceptual synthesis, selection decisions were guided by conceptual objectives and may reflect interpretative positioning inherent to theory-building approaches, while the analysis remains potentially influenced by structural publication bias. Indexed literature tends to overrepresent Western and high-income populations, which may limit the global representativeness of the theoretical integration.

Second, the identification and categorization of “traditional” or “ethnocultural” dietary models is methodologically complex. Cultural food systems are heterogeneous, historically layered, and continuously reshaped by globalization, migration, and the nutrition transition. Any attempt to treat traditional diets as stable entities risks oversimplification. Operationalizing these constructs in empirical research requires careful contextualization and differentiation between historically rooted practices and contemporary hybridized or commercialized patterns.

Third, measuring cultural influences on biological ageing presents substantial practical and epistemological challenges. Reliable quantification of culturally embedded dietary behaviors, alignment of ethnographic observation with biological ageing biomarkers (e.g., epigenetic age acceleration, metabolomic profiles, microbiome diversity), and adequate adjustment for socioeconomic and healthcare variables demand longitudinal and mixed-method study designs that extend beyond the scope of the present conceptual analysis.

Fourth, there is a potential risk of cultural romanticization. Traditional dietary systems evolved under diverse historical conditions, including food scarcity, infectious disease burden, and limited life expectancy. Their association with contemporary longevity cannot be assumed to reflect universal adaptive optimization. Selective emphasis on favorable examples may obscure structural inequalities, micronutrient deficiencies, or adverse health outcomes observed in certain populations maintaining traditional food systems.

Finally, the translation of ENuG-informed insights into clinical or public health practice is constrained by structural determinants such as urbanization, labor market transformation, food industrialization, economic accessibility, migration patterns, and individual metabolic variability. Dietary practices are embedded within complex social and economic systems that may limit the feasibility of implementing culturally grounded nutritional strategies.

Despite these limitations, the interdisciplinary synthesis presented here offers a coherent conceptual platform for future empirical exploration and validation. ENuG should therefore be understood as a theory-building analytical framework intended to stimulate hypothesis-driven research at the intersection of culture, nutrition, and the biology of ageing.

Results

Theoretical Foundations

Modern gerontology is evolving as a multidimensional discipline that seeks to explain not only the biological mechanisms of ageing but also the social, cultural, and ecological determinants shaping individual and population life-course trajectories. Yet, a certain fragmentation of knowledge persists: biomedical gerontology focuses on cellular and molecular processes, social gerontology on institutional and intergenerational interactions, and cultural gerontology on symbolic models of old age and the perception of ageing [14-16]. Nutrition serves as a universal mediator between biology and culture but is rarely considered as an autonomous cultural-biological mechanism of ageing [17].

In recent decades, attention to the role of nutrition in the biology of ageing has increased substantially. A new discipline - nutrigerontology - has emerged, systematically studying how macro- and micronutrients, caloric intake, and diet quality affect key signaling pathways associated with longevity: mTOR, IGF-1, AMPK, and the SIRT system, as well as processes of cellular senescence, inflammation, and mitochondrial dysfunction [18,19]. Nutrigerontology has demonstrated that diet is not merely a behavioral factor but a regulatory element of metabolic control over ageing, influencing the organism’s molecular clocks [20]. However, most studies in this field remain universalist in orientation [21,22]. The diets of northern peoples, tropical communities, or high-mountain populations represent not merely culinary traditions but elements of biocultural adaptation consolidated through evolution. The universalisation of dietary models without accounting for ethnocultural context may lead to the loss of adaptive advantages and the reduced effectiveness of nutrigerontological recommendations across populations.

In the humanities and anthropological sciences, a parallel field has emerged - ethnonutrition (or nutritional anthropology) - which investigates nutrition as a cultural-ecological system. It analyses how cultural norms, beliefs, and social structures determine eating behavior, food taboos, and methods of preparation [23,24]. Ethnonutrition emphasises that diet cannot be considered outside its cultural context, as it reflects collective identity and a society’s adaptation to its environment. Yet, research in this area has largely focused on food security, the transformation of diets under the influence of urbanisation and migration, rather than on ageing as a physiological process [25]. ENuG is therefore proposed as a field of knowledge exploring how culturally specific dietary models affect the biological mechanisms of ageing and human functional resilience.

Another related field - ethnogerontology - studies cultural and ethnic differences in ageing, perceptions of old age, the roles of older people, and intergenerational models of support [26,27]. Ethnogerontology convincingly demonstrates that ageing is not only a biological but also a cultural phenomenon, dependent on the meanings and social roles attributed by society to later life [28]. However, in most cases, it does not include nutritional aspects - it does not examine how cultural dietary patterns affect the physiological mechanisms of ageing.

Thus, there are three key but weakly integrated domains in modern science: biomedical (nutrigerontology), cultural-ecological (ethnonutrition), and sociocultural (ethnogerontology). Between them lies an uncharted area where nutrition, culture, and ageing interact but still are not analytically unified. It is precisely within this intersection that the need arises for a new concept integrating the biological, cultural, and ecological dimensions within a single theoretical framework.

Nutrition represents a distinctive interface linking biology, culture, and behavior. It is a source of metabolic information, a form of social communication, and a practice that ensures adaptation [29]. Through nutrition, humans engage with both natural and cultural environments; it defines life rhythms and relationships with one’s body, family, and community. Therefore, within a gerontological context, nutrition should be viewed as the central channel for integrating biological and cultural processes [30]. Across different cultures, nutrition embodies the idea of the life cycle: it accompanies humans from birth to death, shapes the symbolism of celebration, fasting, and mourning, and creates intergenerational continuity. In this sense, diet represents not only a set of foods but a cultural metaphor for life itself [31].

Contemporary biological studies of ageing demonstrate that key regulatory processes - inflammation, oxidative stress, microbiome composition, and mitochondrial function - are extremely sensitive to the composition, caloric content, and rhythm of eating [19,4]. This provides the basis for integrating perspectives: culture shapes diet, diet modulates biology, and biology determines ageing outcomes. If nutrigerontology answers the question “how does nutrition influence ageing”, ethnonutrition responds to “how does culture shape diet”, and ethnogerontology explores “how does culture shape attitudes to ageing”, the new framework should unify these axes into a single causal sequence: Culture - Nutrition - Biological Mechanisms - Ageing. Such an integrative approach explains why universal dietary models are not equally effective across ethnopopulations. Epidemiological observations indicate that populations maintaining traditional diets often exhibit slower functional ageing even at lower income levels [22,32]. Conversely, the rapid shift to industrialised diets is associated with an increase in metabolic syndromes, obesity, and accelerated biological ageing [25,33].

Accordingly, there emerges a need for a new integrative discipline - ENuG - which unites biomedical and cultural approaches by viewing nutrition as a cultural-biological regulator of ageing rates. The principle of ENuG posits that ethnocultural dietary models are the result of long-term adaptation optimising the interaction between the organism and its environment, while their loss leads to metabolic maladaptation and the acceleration of ageing [34-36].

Thus, through ENuG, ageing can be considered a biocultural process in which nutrition serves as a mediator between culture and physiology. Within this triad, culture provides meanings and behavioral models, nutrition translates these meanings into biological reality, and biology materialises adaptation. It is precisely at this intersection that individual and population ageing trajectories are formed - defining the need for a new interdisciplinary framework for further research and practical applications.

Development and Concept of ENuG

The development of gerontological research in the 21st century increasingly demonstrates that biological ageing cannot be fully understood outside the context of cultural and ecological determinants. The concept of a “universal ageing trajectory” has lost its relevance, as data from large cohort and comparative studies reveal considerable variability in the rates of biological ageing across populations, even under similar medical and economic conditions [37,38]. This variability reflects not only genetic differences but also cultural patterns of diet, physical activity, family structure, and perceptions of old age.

The proposal of ENuG as a distinct analytical domain follows three epistemological criteria commonly applied in theory formation: the identification of a stable analytical object not fully operationalized in existing frameworks (nutrition as a culturally encoded biological regulator of ageing); the formulation of a coherent explanatory model linking previously separated domains (culture → diet → molecular ageing mechanisms → functional resilience); the capacity to generate empirically testable hypotheses and measurable indicators. ENuG is therefore not introduced as disciplinary inflation, but as an effort to formalize a previously implicit intersection into a structured analytical framework.

From Biological to Biocultural Ageing

The paradigm of biocultural ageing posits that ageing represents not merely a set of molecular and physiological changes but an adaptive process shaped by cultural norms, symbolic practices, and lifestyles. This idea finds resonance in the concept of cultural embodiment, which suggests that physiological processes become part of cultural experience [39,40]. In the context of nutrition, this approach acquires particular importance. Culture defines what is considered “proper” food, when, where, and with whom eating should occur, and which foods are associated with purity, health, or sanctity. These norms, repeated across generations, establish stable dietary patterns that exert long-term metabolic and epigenetic effects.

There is now convincing evidence that diets shaped within traditional cultures are optimally aligned with the climate, microbiota, sleep rhythms, and physical activity of local populations [41-43]. For instance, traditional diets of Mediterranean, Caucasian, or Andean communities are characterised not only by specific nutrient ratios but also by a persistent cultural logic of moderation, seasonality, and communal eating [44]. These cultural mechanisms prove as significant for longevity as the dietary components themselves. It therefore becomes evident that nutrition is not simply an external factor in ageing but a biocultural system in which traditions and metabolic adaptations form a single dynamic structure [45].

From Universal Diets to Culturally Specific Models of Longevity

Most existing nutritional recommendations in gerontology - such as the Mediterranean, Dietary Approaches to Stop Hypertension (DASH), Mediterranean-DASH Intervention for Neurodegenerative Delay (MIND), or Blue Zones-inspired diets - are based on population studies conducted predominantly in Western societies [1,4,6,21]. These models have demonstrated effectiveness but are not always applicable to other ethnocultural contexts. For example, typical Uzbek, Kazakh, or Indian cuisines differ in their ratios of proteins and fats, carbohydrate structures, and cooking methods, yet have traditionally supported healthy ageing before the onset of rapid urbanisation [46]. This underscores the necessity of adapting dietary recommendations to local cultural and ecological conditions.

Studies of the nutrition transition show that the replacement of local foods with industrial, processed, and hypercaloric products is accompanied by molecular indicators of accelerated ageing - elevated inflammatory markers, insulin resistance, and altered microbiota composition [25,33,47]. These findings support the thesis that the loss of culturally adapted diets leads not only to an increase in chronic diseases but also to a disruption of biological resilience to ageing. Conversely, traditional dietary models preserving seasonality, locality, and social ritualisation provide a form of geronto-adaptivity - the ability of diet to maintain optimal balance between energy metabolism and restorative processes [22,32]. Thus, the notion of cultural-specific geronto-adaptivity may be viewed as a central link in the new concept.

Ethnonutrigerontology as an Integrative Field

At the intersection of nutrigerontology, ethnonutrition, and ethnogerontology, a theory-building analytical framework is proposed: ENuG. It aims to examine nutrition as the connecting bridge between culture and the biology of ageing, integrating levels of analysis from molecular mechanisms to cultural practices.

ENuG rests upon the following core propositions: Culture determines diet. Eating habits, cooking methods, taboos, and symbolic meanings of foods are formed historically in response to climatic, religious, and ecological conditions. They codify adaptive models influencing physiological stability and health [23]. Diet modulates biological pathways of ageing; nutrition regulates signaling cascades associated with metabolism, inflammation, cellular stress, and senescence: mTOR, AMPK, SIRT1, IGF-1, and the microbiome; biological mechanisms determine functional resilience [19,48]. The level of physical, cognitive, and emotional capacity in older age reflects the efficiency of biocultural adaptation rather than medical intervention alone [38,49].

Thus, ENuG formalizes this multilevel sequence into a structured analytical model linking culture, diet, biological mechanisms, and functional outcomes. This model may serve as a conceptual basis for developing biocultural indices of ageing. At the intersection of ethnocultural, ecological, biomedical, and gerontological dimensions emerges a zone of biocultural equilibrium, where nutrition functions as a mechanism of adaptation, ensuring optimal correspondence between the external environment and internal physiological processes.

The New Role of Traditional Diets in the Context of Ageing

In formulating this new field of knowledge, ENuG reinterprets traditional diets not as “archaic” or “folkloric” elements but as outcomes of long-term cultural adaptation processes, which may have incorporated ecologically advantageous practices over time. For example, the Okinawan diet, characterised by low glycaemic index and high polyphenol content [22]; Georgian and Imeretian cuisines with their emphasis on fermented foods, herbs, and nuts [50,51]; and the Andean diet, rich in plant proteins, amino acids, and antioxidants [52,53]. These nutritional systems ensure high microbiome diversity, moderate caloric intake, optimal fatty acid ratios, and the presence of natural antioxidants - factors associated with slower ageing and reduced risk of chronic disease [44,54].

It is important to acknowledge that the presence of a traditional dietary pattern does not automatically confer favorable health outcomes. In several regions, traditional food systems coexist with micronutrient deficiencies, limited healthcare access, or socioeconomic constraints that negatively affect ageing trajectories. ENuG does not assume inherent superiority of all traditional diets; rather, it proposes that culturally embedded dietary systems may contain adaptive elements whose effects depend on ecological stability, food availability, and broader social determinants of health.

Thus, ENuG provides a theoretical foundation for re-evaluating traditional diets as elements of intangible cultural heritage with demonstrable biomedical value.

Conceptual Distinction of ENuG From Related Disciplines

Unlike nutrigerontology, which is oriented towards universal biological mechanisms, ENuG introduces the cultural-ecological variable as an independent level of analysis. In contrast to ethnonutrition, which concentrates on dietary behavior and social structure, ENuG integrates physiological outcomes and biomarkers of ageing. Finally, unlike ethnogerontology, which addresses cultural meanings of old age, ENuG proposes measurable empirical parameters for assessing the impact of cultural dietary models on biological ageing. In doing so, ENuG establishes a new level of interdisciplinary synthesis in which culture is regarded as an active biological modifier, exerting its influence through nutrition.

While ENuG builds upon existing integrative traditions such as biocultural gerontology and nutritional anthropology, it differs in its analytical focalization. In biocultural ageing research, nutrition is typically treated as one of several environmental exposures. In nutritional anthropology, ageing biology is not a central outcome variable. ENuG introduces a structured causal mediation model in which nutrition functions as the primary operational interface between cultural systems and measurable biological ageing pathways. This explicit mechanistic positioning distinguishes ENuG from broader interdisciplinary approaches that remain conceptually integrative but analytically diffuse.

Contemporary ageing science is shifting from reductionism - the search for a single cause - towards systemic approaches viewing the organism as an open adaptive system. Within this paradigm, nutrition is not only an energy source but also a regulator of information exchange between humans and their environment [55]. ENuG reinforces this logic by suggesting that culturally patterned dietary behaviors may exert cumulative epigenetic influences across generations through shared environments and repeated behavioral transmission. This understanding enables the transition to a new gerontological ontology in which the human being is viewed not in isolation but as a biocultural organism living within a system of symbols, traditions, and metabolic interactions.

While biocultural ageing models conceptualize ageing as the product of biological and sociocultural interactions, they do not typically specify a single operational mediator linking these domains. ENuG advances this perspective by formalizing nutrition as a measurable and biologically active interface through which cultural systems exert material influence on molecular ageing pathways. This operational centering of diet as a mediating mechanism provides analytical specificity and facilitates empirical testing, thereby extending beyond descriptive biocultural integration toward a structured explanatory model.

Thus, ENuG is not merely a new terminological construct but an integrative theoretical platform capable of uniting empirical data on ageing with the cultural and ecological context.

Discussion

ENuG should be understood primarily as a theory-building analytical framework that may evolve into a structured research program. It is not presented as an independent institutionalized discipline in the sociological sense, nor as a standalone methodological protocol. Rather, it functions as a conceptual lens that organizes existing empirical domains under a focused explanatory model centered on nutrition as a biocultural mediator of ageing. In applied contexts, it may inform policy or clinical adaptation strategies, but its current status remains theoretical and hypothesis-generating.

Summarising the data reviewed in the section “Theoretical Foundations”, three key conclusions can be drawn: Nutrigerontology defines the molecular mechanisms of ageing under dietary influence [18-20]; Ethnonutrition reveals the socio-ecological determinants of eating behavior [23-25]; Ethnogerontology demonstrates the cultural variability of attitudes towards ageing [26-28]. ENuG integrates these dimensions, creating a new, biocultural level of analysis in which nutrition functions as a regulator of adaptation between the organism and the cultural environment [29-31,39-41]. A key implication of this integration is the re-conceptualisation of the notion of a “traditional diet”. Within the ENuG framework, it is understood not as an archaic phenomenon but as the result of long-term evolution of cultural practices that ensure physiological stability across populations [22,32,44]. Examples from the Okinawan, Caucasian, and Andean diets illustrate that their geronto-adaptive potential lies not only in the chemical composition of food but also in cultural mechanisms of moderation, communal eating, and ritualised mealtimes [44,50-53]. These traditions shape dietary rhythms and social models that directly influence inflammatory, hormonal, and metabolic pathways associated with longevity [19,38,47].

Accordingly, ENuG allows nutrition to be considered as a cultural-epigenetic system in which symbols and habits consolidate behavioral and physiological adaptations. This concept complements and expands the nutrigerontological paradigm by incorporating cultural and ecological variables.

In contrast to existing models (DASH, Mediterranean, Blue Zones), the ENuG approach emphasises the local relevance of recommendations, thereby increasing both their effectiveness and social acceptability [1,4,46]. Particularly significant is the proposal of ENuG as a research platform. It can serve as the foundation for empirical projects examining the relationships between cultural dietary patterns, biomarkers of ageing (DNA methylation, microbiota, metabolomics), and functional resilience in older adults. Such an approach aligns with the principles of biocultural ageing and may form part of integrated programmes linking public health, sociology, anthropology, and sustainable nutrition [13,19,56-61]. In the longer term, ENuG may contribute to the re-evaluation of nutrition as a component of intangible cultural heritage with medical and social significance [62,63]. This would align the goals of cultural preservation with those of public health, fostering a new paradigm of culturally sensitive longevity.

The formation of the ENuG concept thus opens new horizons for integrating biomedical and cultural sciences in addressing one of the central challenges of modern society - ensuring healthy ageing under conditions of global demographic transformation [56].

Potential for Clinical Practice and Individualised Nutrition

At its current stage, ENuG should be considered a conceptual orientation rather than an immediately applicable clinical model. Any translation into practice would require empirical validation across diverse populations and controlled study designs. Conventional dietary recommendations are often based on averaged data obtained from Western cohorts and therefore may not be sufficiently effective for ethnically diverse populations. ENuG may serve as an alternative - an individualised, culturally sensitive approach to the nutrition of older adults.

The application of ENuG in clinical gerontology would allow the development of personalised nutrition plans that take into account ethnocultural background, habitual tastes, and metabolic characteristics [57]. It would also enable dietary adjustments in accordance with climatic and seasonal factors, which is particularly relevant in regions with extreme temperatures or high altitudes. Furthermore, adherence to dietary therapy could be improved by incorporating elements of familiar culinary practice and the symbolic significance of food [24].

If empirically supported, ENuG-informed approaches may contribute to the prevention of age-related diseases (sarcopenia, type 2 diabetes, atherosclerosis) but also to the restoration of a patient’s cultural identity - an important aspect of psychological and emotional well-being in later life.

Contribution to Public Health and Active Ageing Policy

At the systemic level, ENuG could become a scientific basis for healthy ageing programmes adapted to specific ethnocultural contexts. Within the WHO Decade of Healthy Ageing (2021-2030) framework, the need for multisectoral approaches combining medicine, culture, education, and social policy has been strongly emphasised [58]. Implementing ENuG at the population level would make it possible to identify adaptive elements of traditional diets that could be preserved and integrated into national nutrition strategies; to mitigate the effects of the “nutrition transition” and the growing prevalence of chronic diseases associated with food industrialisation [25]; and to develop intersectoral policies bridging healthcare, culture, agriculture, and education. For example, in the countries of Central Asia and the Caucasus, the revival of traditional dietary patterns - fermented dairy products, seasonal vegetables, herbs, nuts, and portion moderation - may serve not only as an element of cultural revitalisation but also as a public health strategy. Comparable initiatives are already being implemented in Japan, Italy, and Costa Rica under the Blue Zones Projects [22,32,59].

ENuG as an Interdisciplinary Research Platform

From a scientific perspective, ENuG can become a domain in which nutrition is examined not in isolation but as an element of a biocultural ecosystem. Such an approach has the potential to unite researchers from diverse fields - from gerontology and nutrition science to ethnography, sociology, and sustainable food systems.

To facilitate empirical application, the concept of “geronto-adaptivity” may be operationalized through multidimensional indicators combining: Biological markers (e.g., epigenetic age acceleration, inflammatory profiles, metabolic flexibility, microbiome diversity); Dietary structure variables (e.g., degree of processing, dietary diversity indices, meal timing regularity, fermented food consumption); Cultural embedding indicators (e.g., commensality frequency, intergenerational food transmission, seasonality adherence). A preliminary empirical framework would examine whether culturally embedded dietary patterns correlate with slower biological ageing markers after adjustment for socioeconomic and healthcare variables. Such operationalization may enable ENuG to move from conceptual integration toward empirically testable hypotheses.

Key directions for future research may include: investigation of the relationships between traditional diets and biomarkers of ageing, including epigenetic clocks and metabolomic profiles; assessment of the impact of the loss of culinary heritage on the pace of biological ageing in urbanised and migrant communities; creation of an international database of ethnocultural dietary models that integrates biomedical and ethnographic parameters. ENuG could also be incorporated into existing global projects such as the Global Burden of Disease (GBD) and NHANES, with the aim of correlating ageing biomarkers and cultural dietary patterns across different countries [60,61].

Future Research Priorities

To advance from conceptual formulation toward empirical validation, several concrete research directions are proposed: Longitudinal cohort studies examining associations between culturally embedded dietary patterns and biomarkers of biological ageing (e.g., epigenetic age acceleration, inflammatory indices, metabolic flexibility); Mixed-method investigations integrating ethnographic analysis of food practices with quantitative dietary and biomarker assessment; Comparative cross-cultural studies evaluating whether similar dietary components exert differential ageing effects across distinct sociocultural contexts; Intervention-based pilot studies testing culturally adapted nutritional strategies in older adults while adjusting for socioeconomic and healthcare variables.

These priorities aim to move ENuG from theoretical integration toward testable and context-sensitive empirical models.

## Conclusions

ENuG is proposed as a theory-building integrative framework combining the achievements of nutrigerontology, ethnonutrition, and ethnogerontology within a unified biocultural paradigm of ageing. Its central premise is that nutrition is not merely a biological factor but a cultural-biological regulator of ageing, through which human adaptation to the environment is realised.

ENuG provides a framework for viewing ageing as the outcome of interactions between culture, diet, and physiology; for using traditional dietary models as resources for the prevention of age-related diseases; and for designing culturally specific programmes of healthy ageing and sustainable development.

The scientific value of ENuG lies in the creation of a theoretical platform for the study of biocultural ageing trajectories, while its practical significance resides in the potential for implementing personalised and culturally sensitive approaches to nutrition in older populations.

In the long term, ENuG may contribute to the further development of integrative biocultural gerontology by providing a focused explanatory model centered on nutrition as a mediating mechanism. Rather than offering prescriptive solutions, it proposes a conceptual platform for investigating how culturally patterned dietary behaviors interact with biological processes underlying functional resilience in later life.
